# The Osteocyte Transcriptome Is Extensively Dysregulated in Mouse Models of Osteogenesis Imperfecta

**DOI:** 10.1002/jbm4.10171

**Published:** 2019-02-11

**Authors:** Sarah M Zimmerman, Milena Dimori, Melissa E Heard‐Lipsmeyer, Roy Morello

**Affiliations:** ^1^ Department of Physiology and Biophysics University of Arkansas for Medical Sciences Little Rock AR USA; ^2^ Department of Orthopaedic Surgery University of Arkansas for Medical Sciences Little Rock AR USA; ^3^ Division of Genetics University of Arkansas for Medical Sciences Little Rock AR USA

**Keywords:** GENETIC ANIMAL MODELS, COLLAGEN, OSTEOCYTES, OSTEOGENESIS IMPERFECTA, RNASEQ

## Abstract

Osteocytes are long‐lived, highly interconnected, terminally differentiated osteoblasts that reside within mineralized bone matrix. They constitute about 95% of adult bone cells and play important functions including in the regulation of bone remodeling, phosphate homeostasis, and mechanical stimuli sensing and response. However, the role of osteocytes in the pathogenesis of congenital diseases of abnormal bone matrix is poorly understood. This study characterized in vivo transcriptional changes in osteocytes from *Crtap*KO and *oim/oim* mouse models of osteogenesis imperfecta (OI) compared with wild‐type (WT) control mice. To do this, RNA was extracted from osteocyte‐enriched cortical femurs and tibias, sequenced and subsequently analyzed to identify differentially expressed transcripts. These models were chosen because they mimic two types of OI with different genetic mutations that result in distinct type I collagen defects. A large number of transcripts were dysregulated in either model of OI, but 281 of them were similarly up‐ or downregulated in both compared with WT controls. Conversely, very few transcripts were differentially expressed between the *Crtap*KO and *oim/oim* mice, indicating that distinct alterations in type I collagen can lead to shared pathogenic processes and similar phenotypic outcomes. Bioinformatics analyses identified several critical hubs of dysregulation that were enriched in annotation terms such as development and differentiation, ECM and collagen fibril organization, cell adhesion, signaling, regulatory processes, pattern binding, chemotaxis, and cell projections. The data further indicated alterations in important signaling pathways such as WNT and TGF‐β but also highlighted new candidate genes to pursue in future studies. Overall, our study suggested that the osteocyte transcriptome is broadly dysregulated in OI with potential long‐term consequences at the cellular level, which deserve further investigations. © 2019 The Authors. *JBMR Plus* published by Wiley Periodicals, Inc. on behalf of American Society for Bone and Mineral Research.

## Introduction

The skeleton is a dynamic organ that constantly undergoes remodeling to replace old or damaged bone with new, maintain calcium/phosphate homeostasis, and adapt to the external mechanical forces. Three highly specialized cell types are actively involved in the remodeling process: the osteoclasts, which resorb bone; the osteoblasts, which form new bone; and the osteocytes, which sense mechanical stress or damaged bone and orchestrate the remodeling process.^1^, ^2^, ^3^ Osteocytes are former osteoblasts that have been embedded in the newly made bone matrix and eventually became entombed in fluid‐filled lacunae inside the mineralized bone. They are the most numerous (∼95%) cell type in the adult skeleton, and their life span can be as long as that of the bone in which they reside.^1^ Osteocytes extend dendritic cell processes through canaliculi to connect to each other and form an extensive network throughout bone that communicates with blood vessels and at the bone surface with osteoblasts, bone lining cells, and possibly other cells in the marrow.^4^, ^5^, ^6^ Osteocytes sense mechanical stimuli, damaged bone matrix and hormonal signals (for example, parathyroid hormone [PTH], glucocorticoids, and sex steroids). Moreover, osteocytes produce and secrete systemic hormones, such as FGF23, which regulates phosphate homeostasis,^7^ as well as a long list of other signaling molecules that influence osteoblast and osteoclast differentiation, survival, and activity. The list includes bone anabolic WNT signaling molecules (eg, WNT1), inhibitors of the WNT pathway including SOST, DKK1, and SFRP1,^8^, ^9^, ^10^ nitric oxide (NO), and prostaglandin E2 (PGE_2_).^11^, ^12^ In addition, osteocytes play a seminal role in osteoclast generation by virtue of their ability to produce the essential osteoclastogenic factors macrophage colony‐stimulating factor (M‐CSF) and receptor activator of NF‐κB ligand (RANKL) as well as osteoprotegerin (OPG). Notably, mice with an osteocyte‐specific deletion of RANKL are resistant to bone loss induced by unloading,^13^, ^14^ ovariectomy, low‐calcium diet, or glucocorticoid excess.^15^, ^16^, ^17^, ^18^

Although the function of osteocytes in adult bone remodeling and mechano‐sensing is well appreciated, it remains unclear whether this most abundant bone cell type has an active or passive role in congenital diseases of abnormal bone matrix. Osteogenesis imperfecta (OI) is the prototypic inherited disorder of abnormal bone matrix—most often caused by alterations in type I collagen synthesis—associated with a dramatic increase in bone fragility.^19^ Because osteoblasts are the principal bone cell type that synthesizes and secretes large amounts of type I collagen, heretofore the role of osteocytes in OI has been largely overlooked.^20^ Nonetheless, increased osteocyte density has been reported in OI patients, as well as in mouse models of OI,^21^, ^22^, ^23^ and correlates with increasing clinical severity.^21^ Recent evidence that osteocyte dysregulation may be contributing to OI came from the identification of *WNT1* mutations as the cause of early onset osteoporosis or severe OI in patients with mono‐allelic or bi‐allelic *WNT1* pathogenic variants, respectively.^24^, ^25^ Interestingly, the *swaying* mouse model (*Wnt1^sw/sw^*) mimics many typical OI features along with an abnormal osteocyte phenotype and pointed to osteocytes as the potential source of the WNT1 ligand.^26^ Furthermore, the conditional inactivation of *Wnt1* in osteocytes, using Dmp1‐Cre mice, resulted in low bone mass and spontaneous fractures, similar to what was observed in OI patients.^27^ Therefore, osteocytes may contribute to the OI bone phenotype perhaps via altered WNT1 production, but the full spectrum of osteocyte transcriptional changes in OI remains unclear.

In this study, we performed RNA sequencing (RNAseq) from osteocyte‐enriched cortical long bones from control and two mouse models of OI. We used the *Crtap*KO^28^ and the *oim/oim* 22 mouse models which mimic two types of OI with different genetic mutations that result in distinct type I collagen defects but a similarly severe form of OI. We found that the osteocyte transcriptome is significantly altered in both of these models of OI.

## Materials and Methods

### Mice

The *oim/oim* and *Crtap*KO mice were generated previously^22^, ^28^ and were maintained in the C57B6 pure and C57B6/129Sv/ev mixed genetic background, respectively. The wild‐type (WT) control mice used for the RNAseq were littermates of the oim/oim and thus were on a pure C57B6 background. Mice were housed in a pathogen‐free facility with 12‐hour light/dark cycle and unlimited access to water and standard chow diet. All animal work was conducted in accordance to local, state, and US federal regulations. Mice were euthanized at 3 months of age and relevant tissues were harvested according to the recommendations of the Guide for Care and Use of Laboratory Animals (8th edition). The *Crtap*KO mice were genotyped by a PCR protocol using the primers forward 5'‐TGACCGCTTCCTCGTGC‐3' and reverse 5'‐CCCGCCTATCACCAACC‐3' for detecting the mutant allele, and forward 5'‐GGCCAATGACCTCCCGAAG‐3' and reverse 5'‐AACTTCGGGGTAAAGCCAGAG‐3' for the WT allele. The products of the reaction are ∼500 base pairs (bp) for the mutant allele and 180 bp for the WT allele. The *oim* mice were also genotyped by PCR: the primers were 5'‐ACTGTCTGTCTACAGTGAACGTCTTAA T‐3' outer forward, 5'‐GATGTAGATGCATAGAAGACATGGAAGG‐3' outer reverse, 5'‐TTCCCATTTTTTTCTATTATACAGAAACAG‐3' inner forward (WT specific), and 5'‐AATGATTGTCTTGCCCCATTCATTTTTT‐3' inner reverse (oim specific). These four primers were added to the same master mix for a final concentration of 0.1 μM (outer primers) and 1.0 μM (inner primers). The products are 440 bp (all genotypes), 303 bp (WT allele), and 195 bp (oim allele).

### RNA extraction from osteocyte‐enriched bone

See Supplemental Fig. S1 for the workflow of the RNA sequencing experiment. Femora and tibias were harvested from WT, *oim/oim*, and *Crtap*KO mice, *n* = 4/genotype. The mice ID numbers and sex are: WT 3329 female, and 3336, 3337, 3338 males; *oim/oim* 3309, 3414 females, 3413, 3333 males; *Crtap*KO 3324, 3359 males, 3360, 3361 females. Osteocyte‐enriched bone was obtained by removing the epiphyses, thoroughly flushing the bone marrow, and scraping off the periosteal membrane; all these procedures were performed on ice in less than 10 minutes after the initial harvest. The osteocyte‐enriched bone was immediately homogenized in 1 mL of TriPure Isolation Reagent (Roche, Mannheim, Germany; ref 11667157001) and processed for RNA extraction according to the manufacturer's instructions until the chloroform separation step. An equal volume of freshly made, RNase‐free 75% ethanol was added to the clear supernatant obtained from the chloroform separation, mixed, and then transferred to an RNeasy Micro column (Qiagen, Valencia, CA, USA; cat# 74004). Digestion with DNase I and elution of RNA was performed according to the manufacturer's instructions. RNA samples were tested for quality using an Agilent 2100 Bioanalyzer (Agilent Technologies, Santa Clara, CA, USA). Only RNA samples with an RNA integrity number (RIN) of ≥7.0 were accepted for sequencing. The RNA samples that passed the quality test (*n* = 4/genotype) were sequenced and a portion of each sample was saved for real‐time PCR.

### Library preparation, RNA sequencing, and data quality control

Sequencing libraries were prepared from the RNA samples by use of the TruSeq mRNA Sample Prep Kit (Illumina, San Diego, CA, USA). Briefly, total RNA (1 μg) was subjected to polyA selection, then chemically fragmented and converted to single‐stranded cDNA using random hexamer‐primed reverse transcription. The second strand was then generated to create double‐stranded cDNA, followed by fragment end repair and addition of a single A base on each end of the cDNA. Adapters were ligated to each fragment end to enable attachment to the sequencing flow cell. The adapters also contained unique index sequences that allowed the libraries from different samples to be pooled and individually identified during downstream analysis. Library cDNA was PCR amplified and enriched for fragments containing adapters at each end to create the final cDNA sequencing library. Libraries were validated on the Fragment Analyzer for fragment size and quantified by use of a Qubit fluorometer (Life Technologies, Carlsbad, CA, USA). Equal amounts of each library were pooled for sequencing on the NextSeq 500 platform using a high‐output flow cell to generate approximately 25 million 75‐base reads per sample.

### Data post‐processing

Initial RNA sequencing data analysis was performed using Illumina's software portfolio including the BaseSpace (Illumina) analysis pipeline and the Tuxedo suite consisting of Bowtie, TopHat, and Cufflinks to align sequence reads to the murine reference genome GRCm38, identify splice isoforms, and estimate transcript abundance.^29^, ^30^, ^31^

### Quantitation, differential expression analysis, and statistics

Quantification, quality control, and differential expression analysis of the RNA sequencing data were performed using SeqMonk software (version 0.31.1), which runs R‐based statistical analyses and uses Bioconductor packages (eg, DESeq2). The quantification returns values with units of fragments/reads per kilobase million fragments/reads (FPKM/RPKM). To assess the integrity of the sequencing data, a few quality‐control (QC) plots were generated using SeqMonk. The first was the RNA‐Seq QC Plot, which describes the alignment of the sequencing reads (Supplemental Fig. S2*A*). For this study, ideal values for this plot would be 100% of reads aligned “in genes” and nearly 100% “in exons” because we enriched for mRNA, 0% “in rRNA” because ribosomal RNA (rRNA) should have been eliminated, 0% “in MT” because SeqMonk was set to ignore mitochondrial (MT) genes, and 50% “on sense strand” because the sequencing was not strand‐specific. All samples in this study showed similar results, meaning that all samples had a similar composition and were therefore comparable. Next, a box‐and‐whiskers plot of quantitated values for all transcripts in each sample showed that every sample had a similar distribution of expression values, indicating a similar sample composition (Supplemental Fig. S2*B*). Finally, the DataStore Tree plot groups samples based on a “similarity” index calculated from the Pearson correlation coefficient (Supplemental Fig. S2*C*). Notably, there were two samples (*oim/oim* mouse 3309 and *Crtap*KO mouse 3324) that did not appear as closely related to the other OI samples; however, these two were still separate from the WT cluster. Differential expression analysis was set to identify transcripts that had changed by at least twofold and that had a *p* value of <0.05, after adjusting for multiple comparisons. The fold‐change cutoff of ≥2 was fairly stringent in order to reduce the number of false positives.

### Functional annotation and pathway analysis

Biologically relevant patterns of differentially expressed transcripts and altered signaling pathways were identified using the Biological Network Gene Ontology (BiNGO) plugin and CytoScape software (BiNGO, CytoScape)^32^ or the Database for Annotation, Visualization, and Integrated Discovery (DAVID) Bioinformatics Resources v6.8 (DAVID).^33^, ^34^ Additional bioinformatics tools available at the WEB‐based GEne SeT AnaLysis Toolkit (WebGestalt)^35^, ^36^ included the transcription factor activity analysis, which uses the molecular signatures database (MSigDB).^37^

### Real‐time PCR

The reserved portion of the osteocyte‐enriched RNA samples was used to make cDNA with the Transcriptor First Strand cDNA Synthesis Kit (Roche, ref 04379012001), according to the manufacturer's instructions. Real‐time PCR was performed to measure *Mmp2*, *Cdkn1a*, and *Serpine1* gene expression using the ΔΔCT method and normalizing to the geometric mean of five housekeeping genes: *Gapdh*, *G6pdh*, *Pbgd*, *B2mg*, and *Hprt*. The primers used for real‐time PCR are listed in Supplemental Table S1.

## Results

### RNA sequencing results

**Figure 1:**
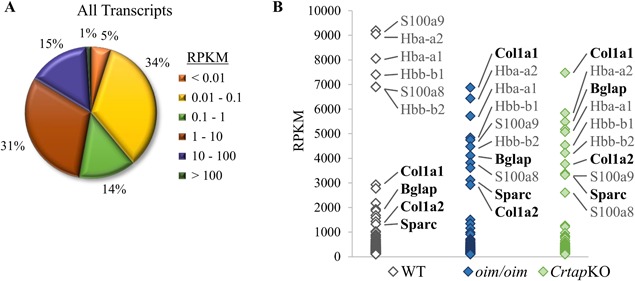
RNA sequencing results. (*A*) The pie diagram illustrates the percent of transcripts at different expression levels in WT osteocyte‐enriched bone for all annotated transcripts in the reference genome. The 25,965 total transcripts are categorized by RPKM value, as detailed in the figure. The percent of total transcripts within each category is displayed next to the associated pie slice. (*B*) The graph displays transcripts with high expression (>100 RPKM) in osteocyte‐enriched bone (267 transcripts in WT, 247 in *oim/oim*, and 212 in *Crtap*KO). Some of the top‐expressing transcripts are noted (bone‐related transcripts in bold). Note that the relative rank of high‐expression transcripts changes for the different groups.

RNA was extracted from femoral and tibial cortical bone of 3‐month‐old WT, *oim/oim*, and *Crtap*KO mice (*n* = 4/genotype, including males and females) as described in the methods and visualized in Supplemental Fig. S1. The RNA was sequenced and the sequencing results from the osteocyte‐enriched bone samples were aligned to a reference mouse genome representing a total of 25,965 unique transcripts. Most transcripts (13,732 or ∼53%) were detected at very low levels, <1 RPKM (reads per kilobase million; Fig. 1 *A*, data from WT group shown). A little less than half of all transcripts had expression levels between 1 and 100 RPKM (11966, or ∼46%), while fewer transcripts (267, ∼1%) were highly expressed at >100 RPKM, and only a handful of transcripts (16, ∼0.06%) had very high expression >1000 RPKM (Fig. 1 *A*). Included in these high‐expression transcripts were the type I collagen genes *Col1a1* and *Col1a2*, as well as the bone‐related transcripts *Bglap* and *Bglap2* (osteocalcin) and *Sparc* (osteonectin). Also included in this group were transcripts for hemoglobin (*Hba‐a2*, *Hba‐a1*, *Hbb‐b1*, and *Hbb‐b2*) and S100 calcium‐binding proteins (*S100a8* and *S100a9*).

The presence of hemoglobin transcripts at such high levels suggested a potential contamination of the osteocyte‐enriched samples by erythropoietic cells. Red blood cells are certainly abundant in the bone marrow but also in small vessels that perfuse the cortical bone.^38^ Although the bone marrow was completely flushed out from the bone shafts, we could not eliminate red blood cells from intra‐cortical vessels, which may explain this result. However, an analysis of cell type–specific gene transcripts indicated that the samples were indeed highly enriched for osteocytes, as osteocyte‐specific genes appeared at much higher levels of expression than non‐osteocyte‐specific genes (Table 1). An alternative source of contamination could be endosteal osteoblasts because these cells adhere to the bone surface and may not be effectively removed by flushing out the bone marrow. Nevertheless, keratocan, a marker highly expressed by osteoblasts but not osteocytes,^39^ was expressed at very low levels in the osteocyte‐enriched samples (Table 1). Although the hemoglobin and S100 calcium‐binding transcripts were the highest expressed transcripts in the WT group, at several thousand RPKM above the type I collagen transcripts, the relative ranks of these transcripts changed in the *oim/oim* and *Crtap*KO groups (Fig. 1 *B*). These changes likely reflect a dramatic upregulation of bone‐related transcripts in the OI mice.

**Table 1:** Expression of Osteocyte and Nonosteocyte Cell Type–Specific Genes

		Expression level (RPKM)		
Gene	Name	WT	*oim/oim*	*Crtap*KO
Osteocyte				
*Bglap*	Osteocalcin	1871.409	4114.907	5482.492^a^
*Sost*	Sclerostin	202.137	143.201	179.367
*Mepe*	Matrix extracellular phosphoglycoprotein	196.191	125.715	184.221
*Dmp1*	Dentin matrix protein 1	136.297	201.513	292.693^a^
Osteoblast				
*Kera*	Keratocan	0.018	0.013	0.017
Osteoclast				
*Oscar*	Osteoclast‐associated receptor	3.216	2.363	2.548
*Dcstamp*	Dendritic cell‐specific transmembrane protein	2.334	1.885	2.115
*Tnfrsf11a*	RANK	1.563	1.236	1.737
*Calcr*	Calcitonin receptor	0.401	0.227	0.408
Hematopoietic stem cells (HSCs)				
*Atxn1*	Sca‐1	1.682	1.679	1.606
*Kit*	c‐kit	7.703	5.728	4.601
*Slamf1*	CD150	2.952	2.236	2.220
*Flt3*	Flk2, CD135	1.842	2.045	1.357
Endothelial cells				
*Pecam1*	CD31	19.287	15.714	17.931
*Sele*	E‐selectin	2.139	1.775	2.006
*Cdh1*	E‐cadherin	0.899	0.937	0.633
Nerve cells				
*Th*	Tyrosine hydroxylase	0.011	0.010	0.010
*Chat*	Choline acetyltransferase	0.009	0.009	0.008

^a^ Denotes differentially expressed genes, ≥2‐fold change and adjusted *p* value <0.05.

### Differential expression analysis

**Figure 2:**
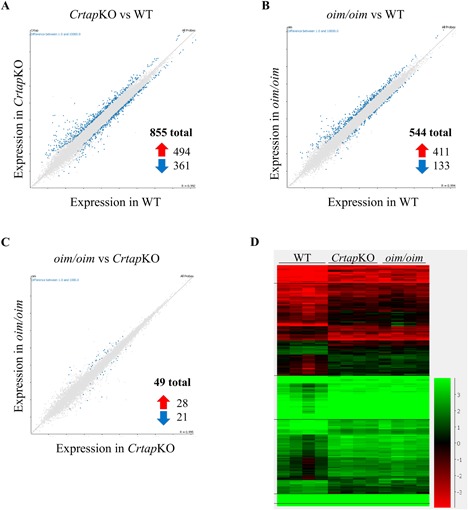
Differential expression analysis comparing osteocyte‐enriched bone in the different genotypes. *Crtap*KO versus WT (*A*), *oim/oim* versus WT (*B*), and *oim/oim* versus *Crtap*KO (*C*) comparisons are visualized in scatter plots of log‐transformed RPKM expression values for every transcript detected in the RNAseq experiment. The *x* axis of each plot shows the average expression in the control group, the *y* axis shows the average in the comparison group, and differentially expressed transcripts are highlighted in blue. The scatter plots were generated in SeqMonk, and annotations were added to indicate which comparison is depicted, axis titles, the total numbers of differentially expressed transcripts, and up‐ and downregulated transcripts (red and blue arrows). In *C*, note that the differentially expressed transcripts are very few and are approximately evenly divided between up‐ and downregulated. (*D*) Expression heatmap of the differentially expressed transcripts shared by both *Crtap*KO and *oim/oim* osteocyte‐enriched bone versus WT. This plot was generated in SeqMonk and annotations were added to indicate the genotypes. The color scale shows RPKM in log‐transformed values, so that negative values (red) indicate low expression, <1 RPKM, values at ∼ 1 RPKM are in black, and higher, >1 RPKM, is in green.

The criteria for a transcript to be considered differentially expressed were arbitrarily set to a fold change of ≥2 and a *p* value <0.05. Our analysis revealed 855 differentially expressed transcripts in the *Crtap*KO group versus WT and 544 differentially expressed transcripts in the *oim/oim* group versus WT (Fig. 2 *A*, *B*). The scatter plots show the differentially expressed transcripts highlighted in blue (all other transcripts are gray), and the distance away from the diagonal line indicates the magnitude of the fold change. The majority of differentially expressed transcripts were upregulated (494 in *Crtap*KO, 411 in *oim/oim*), and the remainder were downregulated (361 in *Crtap*KO, 133 in *oim/oim*) compared with WT (Fig. 2 *A*, *B*, red and blue arrows).

To ascertain differences between the two OI mouse models, an additional differential expression analysis was performed comparing the *oim/oim* group versus the *Crtap*KO group, using the same criteria of fold change ≥2 and *p* value <0.05. This comparison revealed only 49 differentially expressed transcripts (28 upregulated and 21 downregulated, Fig. 2 *C*), suggesting that the osteocyte transcriptome in both models is similarly dysregulated and only small differences exist between the two. For the full lists of the differentially expressed transcripts for *oim/oim* versus WT, *Crtap*KO versus WT, and *oim/oim* versus *Crtap*KO, see Supplemental Table S2. An additional differential expression analysis was performed to test for differences between male and female mice (all genotypes pooled together) in this study. There was only one such transcript: *Ccdc3*, coiled‐coil domain containing 3, which was increased 2.01‐fold in males compared with females.

**Figure 3:**
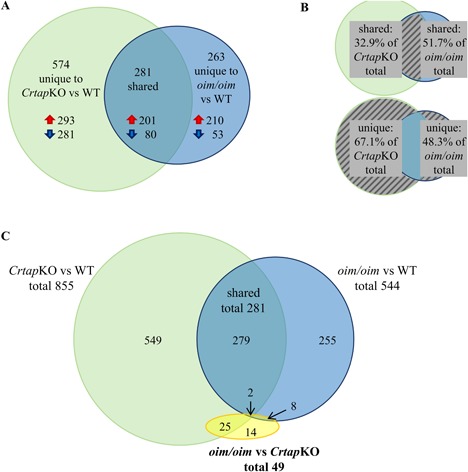
Relationships of differentially expressed transcripts between the genotypes. Area‐proportional Venn diagrams illustrating the differentially expressed transcripts identified in the comparisons *Crtap*KO versus WT (green), *oim/oim* versus WT (blue), and *oim/oim* versus *Crtap*KO (yellow) and the overlaps between them. The numbers of transcripts in each shape or overlap region are noted, and the red and blue arrows indicate the up‐ and downregulated transcripts, respectively. (*B*) These diagrams indicate the percent of differentially expressed transcripts shared by or unique to *Crtap*KO and *oim/oim* versus WT. (*C*) Very few transcripts are differentially expressed between *oim/oim* and *Crtap*KO, as represented by the yellow shape.

Of the total of 855 and 544 differentially expressed transcripts in *Crtap*KO and *oim/oim* compared with WT, 281 were shared by both mouse models (Supplemental Table S3). Remarkably, all 281 trended together, ie, either upregulated in both *oim/oim* and *Crtap*KO or downregulated in both (Fig. 2 *D*). This observation indicates that the two OI mouse models differ from WT in many of the same ways, including bone‐related genes such as *Col1a1*, *Bglap*, *Sparc*, and others. The relationships between the differential expression analyses are depicted using Venn diagrams (Fig. 3). The area of each circle and overlapping region is proportional to the number of transcripts represented by that area. The *Crtap*KO versus WT comparison had a total of 855 differentially expressed transcripts but only shared 281 with *oim/oim* (32.9%, Fig. 3 *A*), indicating that 574 differentially expressed transcripts are unique to *Crtap*KO (67.1%, Fig. 3 *B*). On the other hand, the *oim/oim* versus WT comparison shared 51.7% of differentially expressed transcripts with *Crtap*KO, leaving only 48.3% as unique to *oim/oim* (263 transcripts, Fig. 3 *B*). This suggests that the *Crtap*KO phenotype is more severe than the *oim/oim* phenotype in terms of osteocyte gene expression changes. From the total of 49 differentially expressed transcripts in *oim/oim* versus *Crtap*KO mice, 25 overlapped with the *Crtap*KO versus WT differential expression results, 8 overlapped with *oim/oim* versus WT, and 2 overlapped with the 281 transcripts shared by *Crtap*KO and *oim/oim* (Fig. 3 *C*). That left only 14 transcripts that were differentially expressed uniquely between *oim/oim* and *Crtap*KO. These observations indicate that the two OI models are much more similar to each other than to WT.

### Functional and pathway analysis

**Figure 4:**
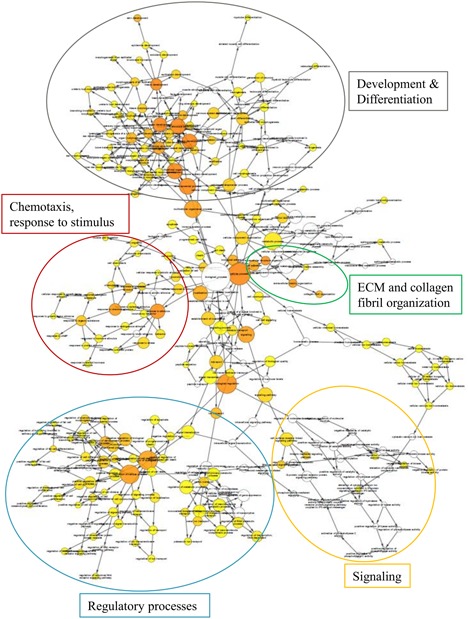
GO terms enriched in the differentially expressed transcripts shared by *oim/oim* and *Crtap*KO groups. This graphic was generated in the BiNGO plugin in CytoScape software, and annotations were added to indicate the clusters of related terms. The nodes each represent a GO term, the size of the node represents the number of transcripts associated with that term, and the color of the node indicates the *p* value (yellow is less significant, orange is more significant). Nodes that are white, or colorless, are not enriched; these are included because they have daughter nodes that are enriched. There are arrows connecting each related node, pointing toward the node that is lower in the hierarchy (look at the pdf version of this image provided in Supplemental Fig. S3, where each node can be more easily visualized).

Next, we performed functional analysis of the 281 differentially expressed transcripts shared by both *Crtap*KO and *oim/oim*, using both the BiNGO and DAVID bioinformatics tools.^32^, ^33^, ^34^ The BiNGO results identified the gene ontology (GO) terms, which were enriched (present more often than would be likely if by random chance) in the shared differentially expressed transcripts. The enriched GO terms and the relationships between them are illustrated as a network in Fig. 4 and Supplemental Fig. S3. The GO terms are organized into clusters of related terms, for example, the largest and densest cluster was related to development and differentiation and included GO terms such as “bone development,” “ossification,” and “skin development” (Fig. 4). Additionally, there were clusters related to chemotaxis, regulatory processes, and a cluster of signaling terms including WNT and integrin signaling. Finally, there was a cluster related to ECM and collagen fibril organization (Fig. 4). Collectively, these results provide insights into the functional changes of osteocytes in the OI mice.

Table 2 lists several other enriched GO terms and the genes from the shared differentially expressed transcript list. The “collagen fibril organization node” is associated with genes like *Adamts14* (a disintegrin and metalloproteinase with thrombospondin repeats 14, ADAMTS14), which is known to cleave collagen;^40^ *Lox* (lysyl oxidase, LOX), which is involved in cross‐linking collagen;^41^ *Serpinh1* (serpin family H member 1, encoding heat shock protein 47, HSP47), which functions as a chaperone for collagen in the ER^42^ and when mutated can cause OI;^43^ and *Col5a1* and *Col5a2* encoding the two α chains of type V collagen, which is important for collagen fibril formation (Table 2).^44^ Some genes are associated with more than one term, such as *Ihh* (indian hedgehog, a signaling molecule) in “extracellular matrix assembly” and “osteoblast differentiation” and *Ibsp*, *Itgb2l*, *Itgam*, and *Adam23* in the terms “cell adhesion” and “integrin‐mediated signaling pathway.” The GO term “oxidation reduction” includes four enzymes that act on collagen (*Lepre1* or P3H1, *P4ha2*, a prolyl‐4 hydroxylase subunit, and the lysyl oxidases *Lox* and *Loxl4*; Table 2).

**Table 2:** Selected Results From BiNGO Analysis of the Shared Differentially Expressed Transcripts in *oim/oim* and *Crtap*KO Versus WT

GO term	*p* Value	Associated genes
Collagen fibril organization	0.000042	*Adamts14, Lox, Col5a2, Serpinh1, Col5a1*
Extracellular matrix assembly	0.007239	*Lox, Ihh*
Collagen metabolic process	0.014645	*Mmp16, Mmp2, Serpinh1*
Osteoblast differentiation	0.048659	*Fgf9, Col1a1, Ihh*
Osteoclast differentiation	0.037989	*Cd300lf, Vit*
Cell adhesion	0.000013	*Ibsp, Nrp2, Hapln1, Col13a1, Adam23, Pdpn, Npnt, Col15a1, Bcan, Cdh2, Col5a1, Itgam, Lama1, Wisp2, Itgb2l, Cgref1, Podxl2, Csf3r, Ihh*
Oxidation reduction	0.000795	*Cyp2u1, Me1, Cyb5r2, Dhrs9, Aldh1l2, Cdo1, Pycr1, Cyp27b1, Lepre1, Tecrl, P4ha2, Aox1, Cybrd1, Loxl4, Lox, Steap1, G6pd2*
Wnt receptor signaling pathway	0.000750	*Fzd9, Wnt1, Wnt4, Nkd2, Sfrp2, Frzb, Apcdd1, Vit*
Integrin‐mediated signaling pathway	0.000747	*Ibsp, Itgb2l, Adamts14, Adam23, Pram1, Itgam*
Negative regulation of Wnt receptor signaling pathway	0.037481	*Fgf9, Sfrp2, Frzb*
Potassium ion transport	0.020904	*Slc12a8, Kcnn1, Scn3a, Kcna1, Kcnh7, Kcnh2*

The color in the *p* value column represents significance, with darker orange meaning more significant *p* values.

The 281 shared differentially expressed transcripts were also analyzed using the DAVID bioinformatics online tools, producing a list of GO terms and KEGG (Kyoto Encyclopedia of Genes and Genomes) Pathways, which were enriched in the shared transcripts. These were arranged into clusters of related terms (the top 6 are reported in Table 3). Three of these clusters were related to the ECM: cluster 1 on ECM components, cluster 2 on ECM organization, and cluster 5 on ECM component interactions with cells and tissues. Some of the results are the same or similar to the BiNGO analysis, such as “osteoblast differentiation,” the many terms related to ECM organization/assembly, “focal adhesion” and “cell adhesion,” and a cluster related to chemotaxis (cluster 4, Table 3). A unique result in the DAVID results was a cluster related to pattern binding, including glycosaminoglycan, carbohydrate, polysaccharide, and heparin binding (cluster 3, Table 3), which may possibly reflect protein interactions in the ECM.

**Table 3:** The Top Functional Annotation Clusters From DAVID Analysis of the Shared Differentially Expressed Transcripts

Annotation Cluster 1	Enrichment Score: 8.001214
GO:0005578∼proteinaceous extracellular matrix	
GO:0031012∼extracellular matrix	
GO:0005576∼extracellular region	
GO:0044421∼extracellular region part	
Annotation Cluster 2	Enrichment Score: 3.995772
GO:0030198∼extracellular matrix organization	
GO:0030199∼collagen fibril organization	
GO:0043062∼extracellular structure organization	
Annotation Cluster 3	Enrichment Score: 3.326833
GO:0005539∼glycosaminoglycan binding	
GO:0030246∼carbohydrate binding	
GO:0001871∼pattern binding	
GO:0030247∼polysaccharide binding	
GO:0008201∼heparin binding	
Annotation Cluster 4	Enrichment Score: 2.663336
GO:0006935∼chemotaxis	
GO:0042330∼taxis	
GO:0007610∼behavior	
GO:0019955∼cytokine binding	
GO:0007626∼locomotory behavior	
GO:0008528∼peptide receptor activity, G‐protein coupled	
GO:0001653∼peptide receptor activity	
Annotation Cluster 5	Enrichment Score: 2.357344
GO:0044420∼extracellular matrix part	
GO:0005201∼extracellular matrix structural constituent	
GO:0043588∼skin development	
GO:0005581∼collagen	
mmu04512:ECM‐receptor interaction	
mmu04510:Focal adhesion	
GO:0008544∼epidermis development	
GO:0007398∼ectoderm development	
GO:0005198∼structural molecule activity	
Annotation Cluster 6	Enrichment Score: 2.054188
GO:0001503∼ossification	
GO:0001501∼skeletal system development	
GO:0060348∼bone development	
GO:0001649∼osteoblast differentiation	

The accession numbers are included; mmu indicates a KEGG pathway.

Another interesting result from the DAVID analysis was an annotation cluster (not in the top 6) related to cell projections, including the GO terms “axon,” “cell projection,” and “neuron projection.” This could likely relate to osteocyte cell projections instead of neuronal projections and might indicate that the formation or connectivity of the osteocyte network is affected in OI. A closer look at the individual genes associated with this cluster revealed several genes thought to play a role during the osteoblast to osteocyte transition, or whose expression was shown to change during the transition (Table 4). Notably, *Pdpn* (podoplanin, also known as E11) may regulate osteocyte morphology and the number of osteocyte projections.^45^ Also remarkable is the fact that almost all of these genes encode plasma membrane proteins, with the exception of PDGFA, which is a secreted signaling factor;^46^ TUBB3, which is a microtubule subunit;^47^ and ERMN (also known as juxtanodin), which binds to the actin cytoskeleton.^48^

**Table 4:** Differentially Expressed Genes Shared by *oim/oim* and *Crtap*KO That Are Associated With the GO Terms Axon, Cell Projection, and Neuron Projection

Gene (ref.)	Name	Fold change in *oim/oim*, *Crtap*KO	Role/function in osteocytes?
*Nrp2* 65, 66	Neuropilin 2	2.06, 2.08	May be decreased in OB‐to‐OT transition
*Cdh2* 67	Cadherin 2	2.34, 2.30	Downregulated during OB‐to‐OT transition
*Pdpn* 68	Podoplanin	2.10, 2.46	Regulates morphology in OB‐to‐OT transition
*Cybrd1* 69	Cytochrome b reductase 1	3.37, 2.21	Unknown
*Trpv4* 70	Transient receptor potential cation channel, subfamily V, member 4	2.34, 2.45	Mechanosensation, also may regulate sclerostin
*Robo2* 71	Roundabout homolog 2	−2.22, −2.77	Unknown
*Met* 72	Met proto‐oncogene	2.14, 3.06	May drive formation of osteosarcoma
*Pdgfa* 73	Platelet‐derived growth factor alpha	2.32, 2.15	Unknown, may be decreased in OB‐to‐OT transition
*Tubb3* 73	Tubulin, beta 3 class III	4.29, 2.64	May be decreased in OB‐to‐OT transition
*Fzd9* 74	Frizzled homolog 9	2.91, 2.18	Presumably as WNT receptor
*Ermn* 48	Ermin, ERM‐like protein	2.31, 2.20	Unknown

The genes are in order of their expression level in the WT group, from the highest, *Nrp2*, at 21.35 RPKM to the lowest, *Ermn*, at 0.062 RPKM, which is practically not expressed.

### Signaling pathways affected

We had hypothesized at the inception of this work that dysregulated signaling pathways in osteocytes may lead to changes in bone homeostasis. To test this hypothesis, we used the transcription factor activity enrichment tool from the online WebGestalt bioinformatics toolset to seek evidence for transcription factor activity in the list of differentially expressed transcripts shared by *oim/oim* and *Crtap*KO. Transcription factors with target genes enriched in the shared transcripts are reported in Table 5 along with the signaling pathway(s) associated with that factor, which were identified by a literature search. WNT signaling was confirmed for three of the results, reinforcing the BiNGO and DAVID analyses. Other potentially affected pathways not suggested by the previous analyses included Hippo, NF‐κB, growth factor, stress, insulin, and hypoxia signaling.

**Table 5:** Select Results of the Transcription Factor Activity Analysis

Transcription factor	*p* Value	Potentially related signaling pathway(s)
Lef1	1.22E‐09	WNT
Lef1	2.19E‐09	WNT
Tcf3	1.44E‐07	WNT
Tef1	6.94E‐07	Hippo
Sp1	1.50E‐06	NF‐κB
Mef2a	1.50E‐06	Growth factors or stress
Foxo4	1.69E‐06	Insulin or hypoxia

Some of the signaling pathways identified by the BiNGO and DAVID analyses were further analyzed. Table 6 shows the expression of genes associated with the GO term integrin‐mediated signaling pathway, which was enriched in the shared differentially expressed genes according to the BiNGO analysis. There were two integrin genes, *Itgam* and *Itgb2l*, both of which were downregulated, and two metalloproteinases, *Adamts14* and *Adam23*, which were upregulated (Table 6). The last two were the upregulated *Ibsp* and downregulated *Pram1*, which encode integrin‐binding sialoprotein and PML‐RARA regulated adaptor molecule 1, respectively (Table 6).

**Table 6:** Genes Related to Integrin‐Mediated Signaling Pathway in the Differentially Expressed Transcripts Shared by *oim/oim* and *Crtap*KO

	Expression (RPKM)			Fold change versus WT	
Gene	WT	*oim/oim*	*CrtapKO*	*oim/oim*	*CrtapKO*
*Itgam*	59.924	27.859	21.747	−2.2	−2.7
*Itgb2l*	50.305	24.078	15.776	−2.1	−3.4
*Adamts14*	4.337	8.983	9.749	2.1	2.3
*Adam23*	0.104	0.400	0.227	3.7	2.2
*Ibsp*	221.734	474.273	510.286	2.3	2.4
*Pram1*	38.375	17.222	17.932	−2.2	−2.1

**Figure 5:**
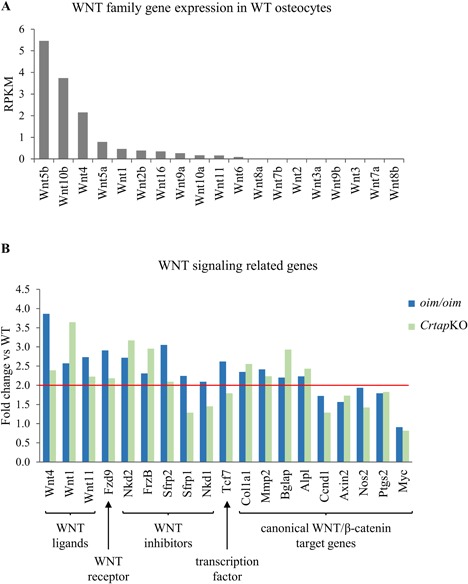
Osteocyte Wnt gene expression and fold change of selected genes related to WNT signaling. (*A*) Graphic representation of all Wnt transcripts expressed by osteocytes in order of abundance, expressed as FKPM. (*B*) The graph displays the fold change versus WT (*y* axis) of several genes (*x* axis) in the *oim/oim* and *Crtap*KO groups (blue and green, respectively). The red line indicates a twofold change. The notations at the bottom indicate the role of the gene product in WNT signaling.

Another of the affected pathway was WNT signaling, indicated in Table 2 by the “Wnt receptor signaling pathway” GO term. The genes associated with that GO term included both WNT ligands and inhibitors, making further investigation of this pathway necessary. A search of WNT‐related genes in all the RNA‐Seq data did not produce any downregulated genes. Interestingly, osteocytes expressed several WNT ligands besides WNT1, and highest levels were detected for *Wnt5b* and *Wnt10b* (Fig. 5 *A*). There were three WNT ligands (*Wnt1*, *Wnt4*, and *Wnt11*) upregulated >2‐fold versus WT (Fig. 5 *B*), although *Wnt11* was not identified as differentially expressed in *Crtap*KO because the *p* value was >0.05. This may be due to the low expression level of *Wnt11* (0.158 RPKM WT, 0.433 RPKM *oim/oim*, and 0.352 RPKM *Crtap*KO). Of note, the two highest‐expressed WNT transcripts in the WT group, *Wnt5b* and *Wnt10b*, were not differentially expressed in the OI models. As shown in Fig. 5 *B*, one WNT receptor was upregulated, Frizzled 9 (*Fzd9*). There were also several WNT inhibitors (*Sfrp2*, *Nkd2*, and *Frzb*), which were upregulated in both OI groups, and *Sfrp1* and *Nkd1*, which were upregulated only in *oim/oim* (Fig. 5 *B*). Additionally, there was one transcription factor that works downstream of WNT signaling that was upregulated in *oim/oim*, *Tcf7* (Fig. 5 *B*). Because both ligands and inhibitors of WNT signaling are upregulated, we sought changes in expression of WNT target genes to determine whether osteocyte WNT signaling increases or decreases. We identified at least four target genes of canonical WNT/β‐catenin signaling that were significantly upregulated (*Col1a1*, *Mmp2*, *Bglap*, and *Alpl*) in both mouse models, while others (*Nos2*, *Ptgs2*, *Axin2, Ccnd1*, and *Myc*) were not differentially expressed.

**Figure 6:**
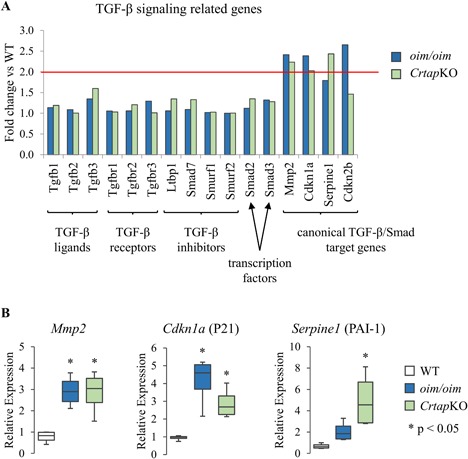
Differential expression of selected genes related to TGF‐β signaling. (*A*) The graph displays the fold change versus WT (*y* axis) of several genes (*x* axis) in the *oim/oim* and *Crtap*KO groups (blue and green, respectively). The red line indicates a twofold change, and the notations at the bottom indicate the role of the gene product in TGF‐β signaling. (*B*) The expression of *Mmp2*, *Cdkn1a*, and *Serpine1* in WT, *oim/oim*, and *Crtap*KO mice was confirmed by real‐time PCR. Expression values are normalized to the geometric mean of five housekeeping genes.

TGF‐β signaling was not among the results of our bioinformatics analysis; however, it is important in bone homeostasis in general and OI in particular.^23^ We therefore probed further into this particular signaling pathway. None of the genes that are directly involved in TGF‐β signaling were differentially expressed in *oim/oim* or *Crtap*KO mice (Fig. 6 *A*), including the three TGF‐β ligands, three TGF‐β receptors, four inhibitors of the signaling pathway (*Smad7*, *Smurf1*, *Smurf2*, and *Ltbp1*), and two transcription factors (*Smad2* and *Smad3*). Importantly, however, four target genes of TGF‐β/Smad signaling were upregulated: *Mmp2* and *Cdkn1a* in both *oim/oim* and *Crtap*KO and *Serpine1* and *Cdkn2b* in just one group (Fig. 6 *A*). The differential expression of three of the TGF‐β target genes was confirmed with real‐time PCR (Fig. 6 *B*). The fourth target gene, *Cdkn2b* (cyclin‐dependent kinase inhibitor 2B, also known as P15), had very low expression levels (<1 RPKM) and was much more difficult to measure by real‐time PCR. Overall, the upregulation of TGF‐β target genes indicates that osteocytes are targets of increased TGF‐β signaling, while lack of differential expression in any TGF‐β ligand suggests that osteocytes are not the source of the increased TGF‐β signal.

## Discussion

Our understanding of the molecular genetics of OI has evolved rapidly in recent years. It is now known that, besides pathogenic variants in either *COL1A1* or *COL1A2*, rarer forms of the disease are associated with bi‐allelic pathogenic variants in a number of different genes that either regulate key steps in type I collagen synthesis pathway or are important for osteoblast differentiation and/or function (for a review, see Marini and colleagues^49^). Consistent with this, the mineral apposition rate (MAR) and bone formation rate (BFR) are significantly low in OI, pointing to the osteoblast as the primary cell type that becomes dysfunctional in this disease. However, even though osteocytes are far more numerous, far more long‐lived, and in more intimate contact with the abnormal matrix than osteoblasts, the role of osteocytes in OI pathogenesis is little, if any, understood. To explore this, we utilized both a gene candidate‐based approach and an unbiased approach. In the former, we hypothesized that osteocytes embedded into an abnormal matrix may become dysregulated and produce higher levels of important cytokines such as RANKL that could contribute, for example, to the high bone turnover observed in OI. We showed that the osteocyte RANKL deletion in *oim/oim* mice completely rescued the loss of cancellous bone mass in these mice, indicating that osteocytes significantly contribute to the low bone mass observed in OI.^50^ In the latter approach and the subject of this study, we set out to compare the transcriptome of osteocytes from control mice with that of osteocytes derived from two mouse models of OI, the *CrtapKO* and the *oim/oim* mouse. We chose *Crtap*KO mice, a well‐characterized mouse model of recessive OI caused by loss of the prolyl 3‐hydroxylation complex and defective type I collagen posttranslational modifications,^28^ and *oim/oim* mice (osteogenesis imperfecta murine), which carry a spontaneous mutation in the *Col1a2* gene, generate α1(I) homotrimers and are commonly used as a model of severe OI.^22^

The analysis reported herein showed that only about 1% of the whole transcriptome of osteocytes is expressed at very high levels (RPKM >100), and importantly, this includes transcripts for both chains of type I collagen. Although it is known that several aspects of the transcriptome and proteome change during the osteoblast‐to‐osteocyte transition,^39^, ^51^ *Col1a1* and *Col1a2* are among the most copious transcripts in both cell types. This observation confirms previous findings from others, showing that type I collagen expression levels are very similar in osteoblasts and osteocytes^39^ and that *Col1a1* is expressed at even higher levels in osteocytes compared with osteoblasts during the early anabolic response to a sclerostin antibody in a rat model.^52^ We show here that type I collagen expression is significantly upregulated in both mouse models of OI (together with *Bglap* and *Sparc*), suggesting perhaps a futile attempt to increase bone formation. ER stress responses to misfolded procollagen chains can contribute to the pathogenesis of OI mouse models.^53^, ^54^ Therefore, upregulation of type I collagen expression in osteocytes suggests that these cells also experience the negative long‐term consequences of ER stress due to misfolded procollagen chains. We did not detect differential expression of factors that mediate the unfolded protein response (UPR), which may suggest an unconventional cell stress response similar to what was described in osteoblasts from the G610C (Amish) mouse model of OI.^54^ These cellular stress responses could be a major force impacting osteocyte transcription and leading to osteocyte dysregulation.

The transcriptome sequencing of the two mouse models of OI used in this study showed that the changes were always consistent, ie, the transcripts were similarly up‐ or downregulated in both models, indicating that distinct alterations in type I collagen can lead to shared pathogenic processes and thus to similar phenotypic/clinical outcomes. Moreover, the gene ontology analysis indicated extensive dysregulation in development and differentiation, regulatory processes, signaling, chemotaxis, and ECM and collagen fibril organization. These data highlight the profound and early changes that impact osteocytes and suggest long‐term consequences at the cellular and tissue level. Further, our gene functional classification with DAVID suggested changes in “cell projections.” Others had shown earlier that during osteocyte development and maturation, there occur gene expression changes in four functional categories: dendritic morphology and canaliculi formation; ECM mineralization and phosphate metabolism; bone formation; and bone resorption.^55^ The results of our analysis confirm that in OI there is a significant dysregulation of transcripts involved in dendrite formation, ECM organization, collagen fibril organization, integrin‐mediated signaling pathway, tissue development, among others. This implies a profound alteration of cellular‐matrix‐tissue interactions that impact cellular function, morphology, and cytoskeletal organization and is consistent with recent evidence of cytoskeletal dysregulation in OI.^56^, ^57^ Of the genes implicated in bone formation, transcripts involved in the WNT signaling pathway were differentially expressed in both our OI models. These included at least 3 WNT ligands but also inhibitors of WNT signaling, suggesting a cellular attempt to increase osteoblast differentiation and function, perhaps triggered by the osteocyte sensing a defective matrix. Interestingly, genes involved in bone resorption (including *Tnfsf11*, *Tnfrsf11a*, *Tnfrsf11b*, *Csf1*, *Csf1r*, *Ctsk*, *Nfatc1*, *Dcstamp*, and *Oscar*) were not differentially expressed compared with WT.

TGF‐β is an important signaling molecule in bone homeostasis and a coupling factor between bone resorption and bone formation.^58^ Mouse models of increased TGF‐β signaling exhibit high bone turnover, low bone mass, and increased osteocyte density;^59^ whereas downregulation of TGF‐β signaling results in decreased bone resorption, high bone mass, and decreased osteocyte density.^60^ Excessive TGF‐β signaling has been recently identified as a seminal pathogenetic mechanism in OI.^23^ Our transcriptome analysis suggests for the first time that osteocytes are also targets of increased TGF‐β signaling. This is consistent with the increased expression of TGF‐β downstream signaling targets in both *Crtap*KO and *oim/oim* mice. However, none of the TGF‐β ligands were upregulated in osteocytes, suggesting that osteocytes are not the source of the TGF‐β. An alternative possibility is that TGF‐β may not be retained in the OI matrix and becomes more bioavailable in the lacuno‐canalicular fluid. Increased TGF‐β signaling in cells of the osteoblast lineage in OI may inhibit osteoblast differentiation^61^, ^62^ but also increase conversion of osteoblasts into osteocytes,^63^, ^64^ which would be consistent with previous findings of increased osteocyte density in OI. Our transcription factor activity enrichment analysis identified new pathways, such as the Hippo and the NF‐κB pathway (Table 5), that may be dysregulated in OI, but their putative role is unclear.

There are several caveats in our study. First, osteocyte dysregulation in the *Crtap*KO mouse seems more variable and/or more severe than in the *oim/oim* mouse, with many more differentially expressed transcripts than *oim/oim* (855 versus 544, respectively). This difference is due to a number of downregulated genes unique to the *Crtap*KO group. This could be accounted for by the fact that the *Crtap*KO mice are maintained on a mixed genetic background, while *oim/oim* are on a pure C57Bl/6 background. Second, bioinformatics tools do not contain the same wealth of annotations related to osteocytes as they do for other, more popularly studied cell types. For instance, a search of geneontology.org for “neuron” returns 647 annotations related to neurons, but a search for “osteocyte” returns zero. Third, our RNA preparation approach excluded microRNAs and therefore this study cannot exclude the possibility that this important class of gene translation regulators are also of relevance to OI. Fourth, the mouse models of OI that we used mimic rare forms of OI in the human population and thus it is possible that classic, autosomal dominant mutations in type I collagen genes (eg, Gly substitutions) may result in different alterations of the osteocyte transcriptome.

In conclusion, the osteocyte transcriptome is greatly dysregulated in OI, including the WNT and TGF‐β signaling pathways. We think that this work suggests many interesting potential directions for future research into the unrecognized role of osteocytes in OI.

## Disclosures

All authors state that they have no conflicts of interest to disclose.

## Supporting information

Supporting Figure S1.

Supporting Figure S2.

Supporting Figure S3.

Supporting Table S1.

Supporting Table S2.

Supporting Table S3.
