# Supplementary figures and images for: The Osteocyte Transcriptome Is Extensively Dysregulated in Mouse Models of Osteogenesis Imperfecta
Source: JBMR Plus. 2019 Feb 11;3(7):e10171. doi: 10.1002/jbm4.10171 (PMC6659450; doi:10.1002/jbm4.10171)

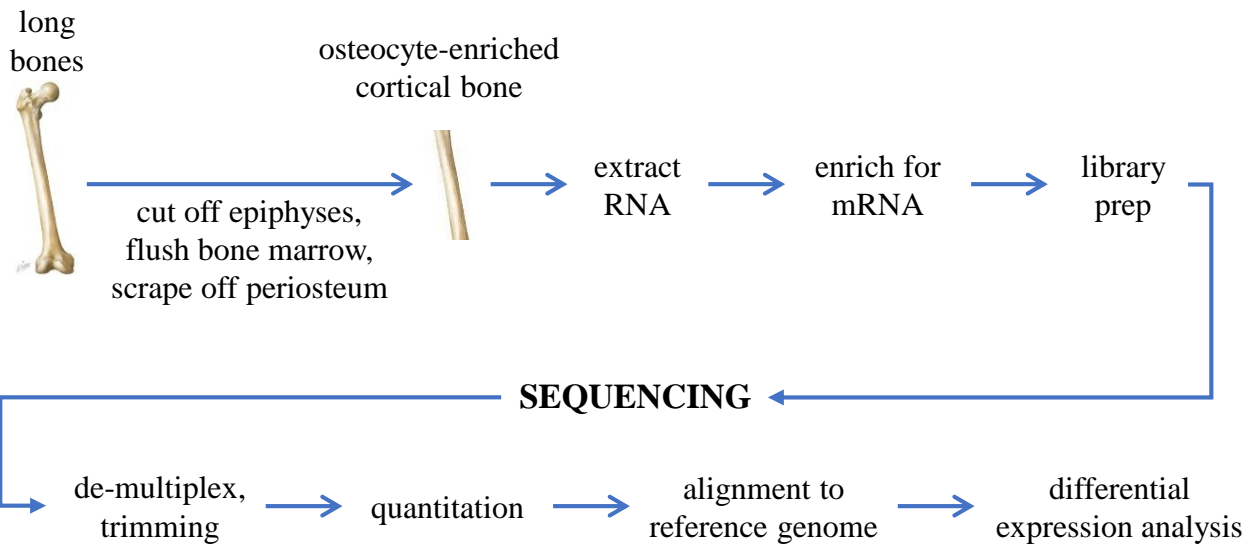

Supplement: Supplementary file 1 — Supporting Figure S1. [file JBM4-3-na-s001.pdf]

A

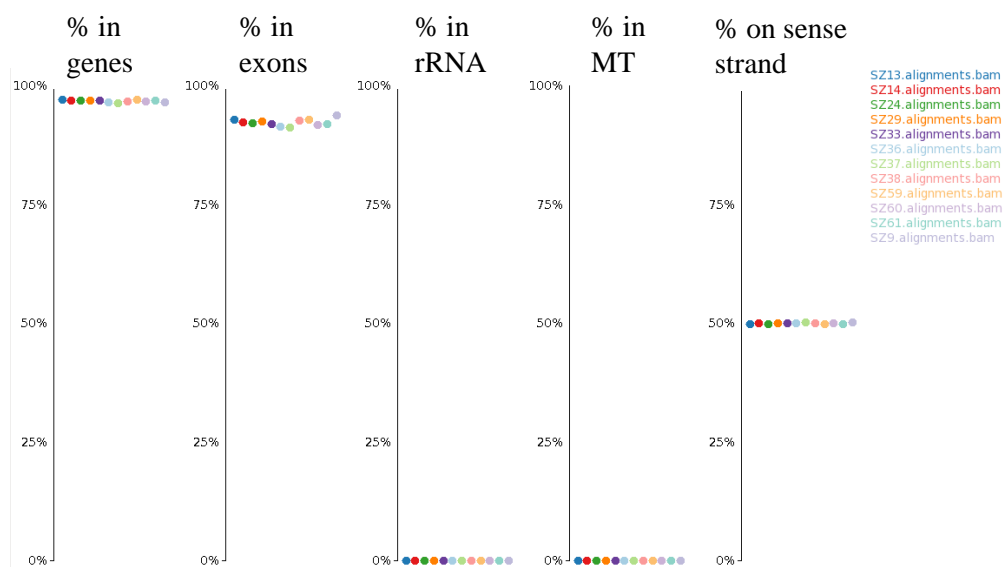

B

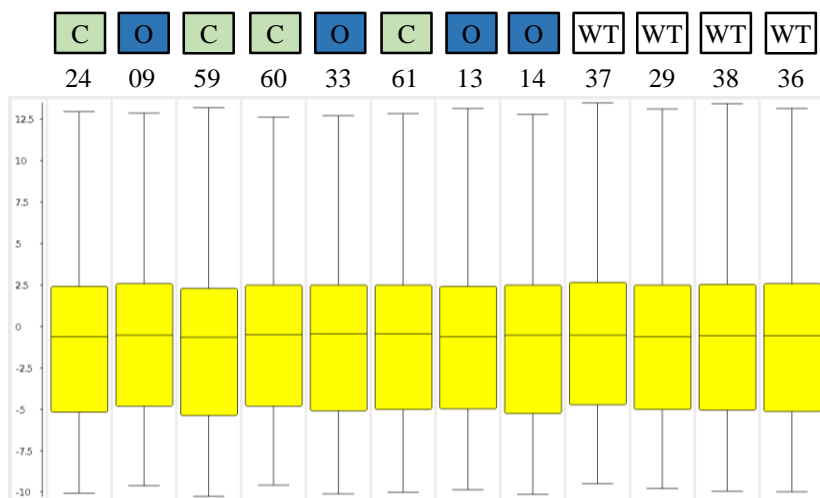

C

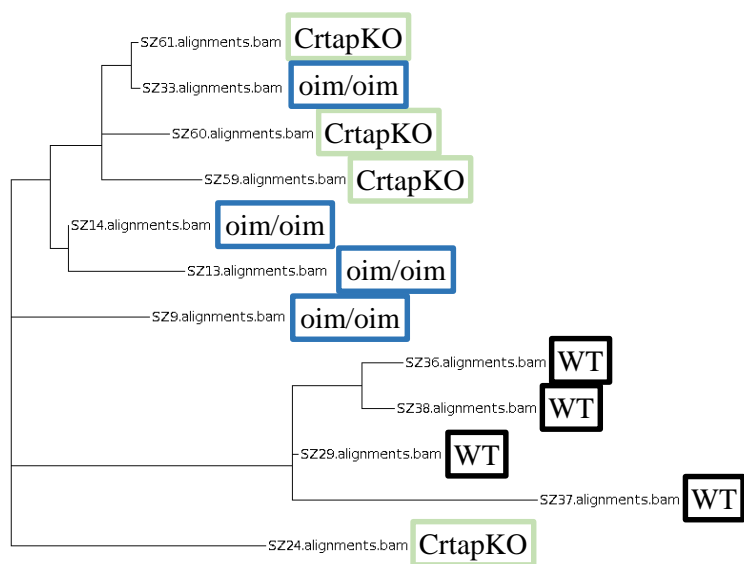

Supplement: Supplementary file 2 — Supporting Figure S2. [file JBM4-3-na-s002.pdf]

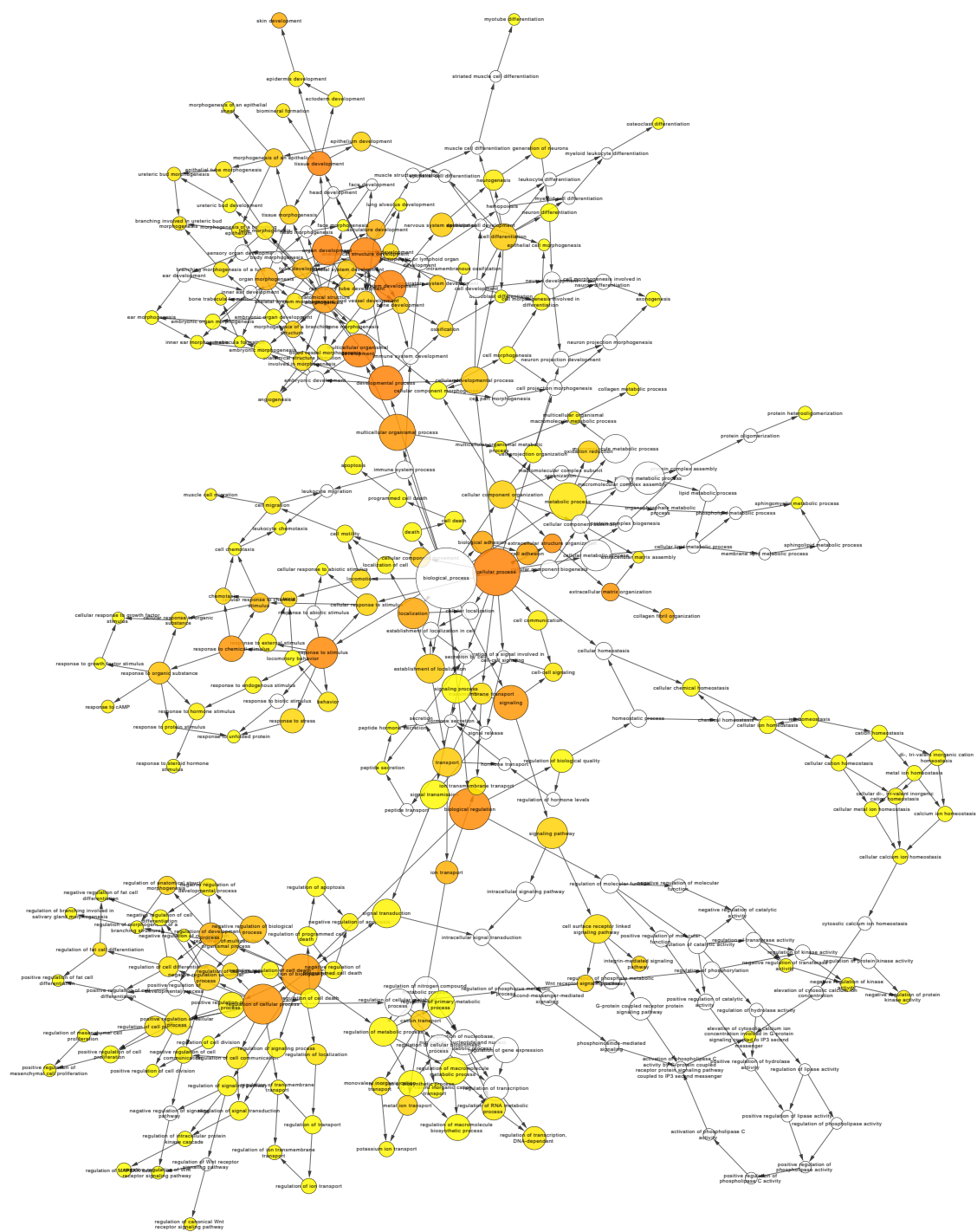

Supplement: Supplementary file 3 — Supporting Figure S3. [file JBM4-3-na-s003.pdf]
