# Supplementary material for: The Osteocyte Transcriptome Is Extensively Dysregulated in Mouse Models of Osteogenesis Imperfecta
Source: JBMR Plus. 2019 Feb 11;3(7):e10171. doi: 10.1002/jbm4.10171 (PMC6659450; doi:10.1002/jbm4.10171)
Supplement: Supplementary file 4 — Supporting Table S1. [file JBM4-3-na-s004.docx]

**Supplementary Table 1. Primers used for real-time PCR.**

| **Gene** | **Primer Sequences 5’ to 3’** |
| --- | --- |
| *Mmp2* | F - GCTCCACTCTTCTGGTTCTTC |
|  | R - CCCTCCTAAGAAAGTCTCTATTA |
| *Cdkn1aI* (P21) | F - CGGAGGAACAGTCCTACTGATA |
|  | R - CAGGTAAGAAGTGGCAAGGAA |
| *Serpine1* (PAI-1) | F - CCTGGTCAACCACCTTAGTTAG |
|  | R - GAGTGGCCTGCTAGGAAATTA |
| *Gapdh* | F - GCAAGAGAGGCCCTATCCCAA |
|  | R - CTCCCTAGGCCCCTCCTGTTATT |
| *G6pdh* | F - AGATTGATCGAGAAAAGCC |
|  | R - AGCTGGGTTTACTGGTG |
| *Pbgd* | F - GTGATGAAAGATGGGCAA |
|  | R - TCTTGGCTCCTTTGTTGA |
| *B2mg* | F - GGTCTTTCTGGTGCTTGTC |
|  | R - CGTATGTATCAGTCTCAGT |
| *Hprt* | F - TCTGGTAGATTGTCGCTTATCTTG |
|  | R - TAGATGCTGTTACTGATAGGAAATCGAG |

F = forward, R = reverse.
